# Surrogate Model Application to the Identification of Optimal Groundwater Exploitation Scheme Based on Regression Kriging Method—A Case Study of Western Jilin Province

**DOI:** 10.3390/ijerph120808897

**Published:** 2015-07-30

**Authors:** Yongkai An, Wenxi Lu, Weiguo Cheng

**Affiliations:** 1 Key Laboratory of Groundwater Resources and Environment, Ministry of Education, Jilin University, Changchun 130021, China; E-Mails: anyongkai1991@163.com (Y.A.); cwg2015@126.com (W.C.); 2 College of Environment and Resources, Jilin University, Changchun 130021, China

**Keywords:** western Jilin province, simulation model, LHS, regression kriging method, surrogate model, optimization model

## Abstract

This paper introduces a surrogate model to identify an optimal exploitation scheme, while the western Jilin province was selected as the study area. A numerical simulation model of groundwater flow was established first, and four exploitation wells were set in the Tongyu county and Qian Gorlos county respectively so as to supply water to Daan county. Second, the Latin Hypercube Sampling (LHS) method was used to collect data in the feasible region for input variables. A surrogate model of the numerical simulation model of groundwater flow was developed using the regression kriging method. An optimization model was established to search an optimal groundwater exploitation scheme using the minimum average drawdown of groundwater table and the minimum cost of groundwater exploitation as multi-objective functions. Finally, the surrogate model was invoked by the optimization model in the process of solving the optimization problem. Results show that the relative error and root mean square error of the groundwater table drawdown between the simulation model and the surrogate model for 10 validation samples are both lower than 5%, which is a high approximation accuracy. The contrast between the surrogate-based simulation optimization model and the conventional simulation optimization model for solving the same optimization problem, shows the former only needs 5.5 hours, and the latter needs 25 days. The above results indicate that the surrogate model developed in this study could not only considerably reduce the computational burden of the simulation optimization process, but also maintain high computational accuracy. This can thus provide an effective method for identifying an optimal groundwater exploitation scheme quickly and accurately.

## 1 Introduction

Simulation and optimization is the main technical method for mechanism analysis, simulation prediction and scheme optimization of actual groundwater systems at present [1,2,3]. In general, the simulation model is used to simulate the actual groundwater system using a numerical method, which can not only express the intrinsic regularity of the actual groundwater system, but also describes the principles and regularities of physics, chemistry and biology followed by the actual groundwater system. As a replica of the actual groundwater system, it can also depict the response relationship between input and output. In a word, the simulation model is applied to predict the results of changes of natural conditions and human activities [4,5].

The simulation model can solve prediction problems in a given decision-making input, but it cannot indicate to us which decision-making input is able to obtain optimal response. Fortunately, the optimization model (operational research model) can solve the optimization problem, by which the optimal decision-making scheme should be obtained by optimizing the decision-making input scheme under the given objectives and constraints.

It should be noted that the optimization model must be based on the simulation model so as to ensure that the optimization process is followed by the intrinsic principle and regularity of the actual groundwater system (presented as simulation model). Therefore, the simulation model needs to be embedded in the optimization model by certain methods to make it become a part of the optimization model. Embedding method, response matrix method and state transition equation method are three methods commonly used to deal with how to embed and invoke the simulation model in the optimization model. However, all of them have their limitations. For example, the embedding method is used to solve small-scale problems generally, but problems of dimension disaster may be produced when using it to solve large-scale problems; the response matrix method is just used to solve linear problems. Comparing these two methods, the computational burden could be reduced to some extent when using the state transition equation method to solve large-scale, multi-period and nonlinear problems, but it still cost a great deal of time to solve the actual problems. Consequently, it is inappropriate for us to use these three methods to solve the large-scale, multi-period and nonlinear groundwater problems [6].

In recent years, some scholars have proposed a surrogate model of the simulation model, where the surrogate model is invoked by the optimization model directly in the process of the iterative solution of the optimization model, which not only solves large-scale nonlinear groundwater problems, but also reduces the huge computational burden and maintains a high computational accuracy. The frequently used surrogate models include the BP neural network model, the RBF neural network model, the regression kriging model, the support vector machine model and so on [7,8,9,10,11], which have been proved to substitute the simulation model. However, researches using the surrogate model of the simulation model to solve the groundwater flow optimization problem in reality are sparse, and both the high computational accuracy and low computational burden need to be verified in the process of the surrogate model invoked by the optimization model. The interpolation results of the regression kriging method have been proved to be very effective because it is unbiased and has minimum estimation variance [12,13], thus the regression kriging method is generally selected in the surrogate model of the simulation model [14,15,16,17,18].

Groundwater is one of the most important water resources for domestic water and agricultural water in western Jilin province, and exploiting groundwater excessively could cause geological environmental disasters including land collapse, soil salinization, desertification and so on. Therefore groundwater should be exploited in a reasonable way in life, which requires the search for an optimal exploitation scheme with consideration of allowable groundwater withdrawal and economic benefits.

This paper selected the western Jilin province as the study area and first established a numerical simulation model of groundwater flow in order to reduce the continuous decline of the groundwater table of Daan county in western Jilin province in recent years. Then four exploitation wells were set in the Tongyu county and Qian Gorlos county respectively so as to supply water to Daan county. Utilizing the LHS method in the above eight exploitation wells to extract 40 groups of exploitation schemes, they were then introduced into a numerical simulation model of groundwater flow so as to obtain a drawdown dataset of the groundwater table. A surrogate model of the numerical simulation model of the groundwater flow was established by the regression kriging method using the above exploitation schemes and drawdown dataset of the groundwater table. Using the LHS method again 10 groups of exploitation schemes were obtained which were introduced into the groundwater flow numerical simulation model and surrogate model simultaneously so as to verify the computational accuracy of the surrogate model. In view of geological environmental disasters on continuous decline of the groundwater table and the difference of the groundwater exploitation costs of the eight exploitation wells, an optimization model was established to search an optimal groundwater exploitation scheme using the minimum drawdown of groundwater table and minimum cost of groundwater exploitation as multi-objective functions. The optimal exploitation scheme was achieved in the process of the surrogate model invoked by the optimization model.

## 2. Study Area and Methods

### 2.1. Study Area

The western Jilin province is located in the southwest of Songnen Plain where it is situated in the transitional zone from semi-humid to semi-arid area. The geographic coordinate lies between 123°09′~124°22′ east longitude and 44°57′~45°46′ north latitude. The whole region is mainly affected by the inland climate of Inner Mongolia and has typical features of the continental climate. The annual average air temperature is 4.6 °C, and the temperature in the southwest is higher, while the north and east are relatively lower. The average annual precipitation in this region is about 400–500 mm, and its temporal-spatial distribution is extremely uneven due to the influences of geographical location and topography.

The study area is a huge aquifer system which has multiple aquifers, including pore unconfined aquifers and pore confined aquifers (shallow and middle-deep), pore-fracture aquifers of Daan and Taikang formations of Neogene (deep), fracture-pore aquifers of lower and upper cretaceous (deep). The recharge sources of groundwater include precipitation infiltration, river leakage, irrigation, infiltration, and lateral groundwater runoff, in which precipitation infiltration is in the dominant position. The discharge of groundwater includes groundwater evaporation, discharge of river, lateral groundwater runoff and artificial exploitation, in which the groundwater evaporation and artificial exploitation are in the dominant position.

### 2.2. Methods

#### 2.2.1. Latin Hypercube Sampling Method

LHS is a kind of homogeneous stratified sampling method, developed from the monte-carlo method [19,20]. The basic principle of this method is to divide the whole sample space into several subintervals, and choose randomly a sample in these subintervals. In this way, the sampling results can cover the entire sample space and will be more representative [21,22,23,24]. The detailed sampling process is described as follows:

Suppose the dimension of random variable is κ, $x^{i}\;\in \left[x_{l}^{i},\;x_{u}^{i}\right]$, i=1, 2, …, k, where $x^{i}$ is the $i^{th}$ variable, $x_{l}^{i}$ and $x_{u}^{i}$ are the lower and upper limits of $i^{th}$ variable respectively. Then the process of stratified sampling for a multi-dimensional random variable is described as follows [25,26,27,28]:

(1) Determining the sampling scale of random variable (*N*).
(2) Dividing each variable into *N* equiprobable intervals, $x_{l}^{i}\;=\;x_{0}^{i}\;<x_{1}^{i}\;<\cdots \;<\;x_{j}^{i}\;<x_{j+1}^{i}\;<x_{N}^{i}\;=\;x_{u}^{i}$, and the probability of each interval is 1/*N*.
(3) Extracting a random sample from each interval of variable *x_i_*, κ refers to variables, and then there are κ×N samples.
(4) The *N* samples extracted respectively from variable *x*^1^ and *x*^2^ are matched randomly without repetition. Then let the matching process go on until the samples extracted from all the variables *x^i^* are completely matched. The eventual matched form is as follows:
  (1) $$X=\left[\begin{array}{l} x_{j}^{1},\;x_{j}^{2},\;\cdots \;x_{j}^{k}\\ x_{j}^{1},\;x_{j}^{2},\;\cdots \;x_{j}^{k}\\ \;\vdots \;\ddots \;\vdots \\ x_{j}^{1},\;x_{j}^{2},\;\cdots \;x_{j}^{k} \end{array}\right]\;,\;j=1,\;2,\;\cdots N$$

#### 2.2.2. Regression Kriging Method

The kriging method is a geostatistics technique which has many different types [29,30,31], and each type has its own special features. The regression kriging method is a type of kriging method, which was first introduced as a surrogate model by Sacks [32,33]. Many researchers now use the regression kriging method to establish the surrogate model.

The form of regression kriging model is [34,35,36,37]

(2) $$y\left(x\right)=\;f^{T}\left(x\right)\beta +z\left(x\right)\;=\sum_{j=1}^{k}f_{j}\left(x\right)\beta_{j}+z\left(x\right)\;$$

where $\sum_{j=1}^{k}f_{j}\left(x\right)\beta_{j}$ is the term of deterministic functions, *β_j_* refers to the coefficients of the deterministic function, and *f_j_*(*x*) is a known regression function of *p*-order, which is usually a polynomial function of 0-order, 1-order, or 2-order.

(3) $$\text{0-order: }p=1,\;f_{1}\left(x\right)=1$$

(4) $$\text{1-order: }\begin{array}{l} p=n+1,\;\\ f_{1}\left(x\right)=1,\;f_{2}\left(x\right)=x_{1},\;\cdots ,\;f_{n+1}\left(x\right)=x_{n} \end{array}$$

(5) $$\text{2-order: }\begin{array}{l} p=\left(n+1\right)\left(n+2\right)/2,\;\\ f_{1}\left(x\right)=1\\ \;f_{2}\left(x\right)=x_{1},\;\cdots ,\;f_{n+1}\left(x\right)=x_{n}\\ f_{n+2}\left(x\right)=x_{1}^{2},\cdots ,\;f_{2n+1}\left(x\right)=x_{1}x_{n}\\ f_{2n+2}\left(x\right)=x_{2}^{2},\cdots ,\;f_{3n}\left(x\right)=x_{2}x_{n}\\ \cdots ,\;f_{p}\left(x\right)=x_{n}^{2} \end{array}$$

*z* (*x*)is a stochastic process with zero-mean, variance σ^2^, and covariance

(6) $$Cov\left[\;z\left(x_{i}\right),\;z\left(x_{j}\right)\;\right]=\sigma_{z}^{2}R\left(x_{i},\;x_{j}\right)$$

where *R* (x*_i_*, x*_j_*)is the correlation function, depending only on the distance vector of x*_i_* and x*_j_*, not on their locations. The common types of correlation functions are as follow [38]:

$$\text{Exponential function: }R\left(x_{i},\;x_{j}\right)=\exp\left[-\sum_{k=1}^{n}\theta_{k}\left|x_{i}^{k}-x_{j}^{k}\right|\right]$$

(7) $$\text{Gauss function: }R\left(x_{i},\;x_{j}\right)=\exp\left[-\sum_{k=1}^{n}\theta_{k}{\left|x_{i}^{k}-x_{j}^{k}\right|}^{2}\right]$$

(8) $$\text{Cubic-spline function: }R\left(x_{i},\;x_{j}\right)=\left\{ \begin{array}{ll} 1-15\xi_{k}^{2}+30\xi_{k}^{3} & for\;0\leq \xi_{k}\leq 0.2\\ 1.25{\left(1-\xi_{k}\right)}^{3} & for\;0.2\leq \xi_{k}\leq 1,\;\xi_{k}=\theta_{k}\left|x_{i}-x_{j}\right|\\ 0 & for\;\;\xi_{k}\geq 1 \end{array}\right.$$

where ξ*_k_* are the unknown parameters, $x_{i}^{k}$ and $x_{j}^{k}$ are the *k^th^* component of sample points x. The Gauss function had been proved feasible in many researches [15,39], thus it was selected as a correlation function in this paper.

For a given set of sample points, $x={\left[x_{1},\;x_{2},\;\cdots ,\;x_{i},\;x_{j},\cdots x_{m}\right]}^{T}$ (each sample point $x_{i}^{k}\left(k=1,\;2,\;\cdots ,\;n\right)$ and the corresponding response $y={\left[y_{1},\;y_{2},\;\cdots ,\;y_{i},\;y_{j},\cdots y_{m}\right]}^{T}$, the prediction of the unsampled point’s response *Y*(*X*) can be represented as a function of the unknown parameters β and *θ_k,_, k=1,2,…,n* [40].

(9) $$Y\left(X\right)=f^{T}\left(X\right)\beta^{*}+r^{T}\left(X\right)R^{-1}\left(Y-f\left(X\right)\beta^{*}\right)$$

The Equation (10) is a general form of regression kriging model, which is established by the following steps:

(1) *r*, the correlation matrix between *m* samples and prediction points *x*, and *R*, the correlation matrix between *m* samples, are calculated by Equation (8).
  (10) $$r\left(x\right)=\left[R\left(x,\;x_{1}\right),\;R\left(x,\;x_{2}\right),\cdots ,\;R\left(x,\;x_{i}\right),\;R\left(x,\;x_{j}\right),\;\cdots R\left(x,\;x_{m}\right)\right]$$
  (11) $$R=\left[\begin{array}{l} R\left(x_{1},\;x_{1}\right)\;\;\cdots \;R\left(x_{1},\;x_{m}\right)\\ \;\;\;\;\;\;\vdots \;\;\;\;\;\;\;\;\;\ddots \;\;\;\;\;\;\;\vdots \\ R\left(x_{m},\;x_{1}\right)\;\;\cdots \;R\left(x_{m},\;x_{m}\right) \end{array}\right]$$
(2) *f* (*X*), referring to the known regression functions of *p*-order, is calculated through equation 5.
  (12) $$f\left(X\right)=f={\left[f_{1}\left(X\right),\;f_{2}\left(X\right),\;\cdots ,\;f_{k}\left(X\right)\right]}^{T}$$
(3) β^*^ is the estimated value of *β*, which is obtained by the generalized least-squares method.
  (13) $$\beta^{*}={\left(f^{T}R^{-1}f\right)}^{-1}f^{T}R^{-1}Y$$
(4) The estimated value of *σ*^2^ is obtained by the following equation.
  (14) $$\sigma^{2}=\frac{1}{m}{\left(Y-f\beta^{*}\right)}^{\text{T}}R^{-1}\left(Y-f\beta^{*}\right)$$
(5) The parameter *θ_k_* is obtained when the following equation achieves its maximum value, and this method is named as the maximum likelihood estimation method. The basic idea of this method (Maximum Likelihood, ML) is that the most reasonable parameter estimator is determined when extracting an n group sample observation value from the sample population of the model randomly and making the n group sample observation value selected from the overall model have a maximum probability.
  (15) $$MLE=-\frac{1}{2}\left(m\ln\left(\sigma^{2}\right)+\ln\left|R\right|\right)$$

#### 2.2.3. Numerical Simulation of Groundwater Flow

The aimed for aquifer located in western Jilin Province is a pore aquifer which is composed of unconfined aquifer and confined aquifer. In the middle of these two aquifers, there is a weakly permeable clayey soil layer.

**Figure 1:**
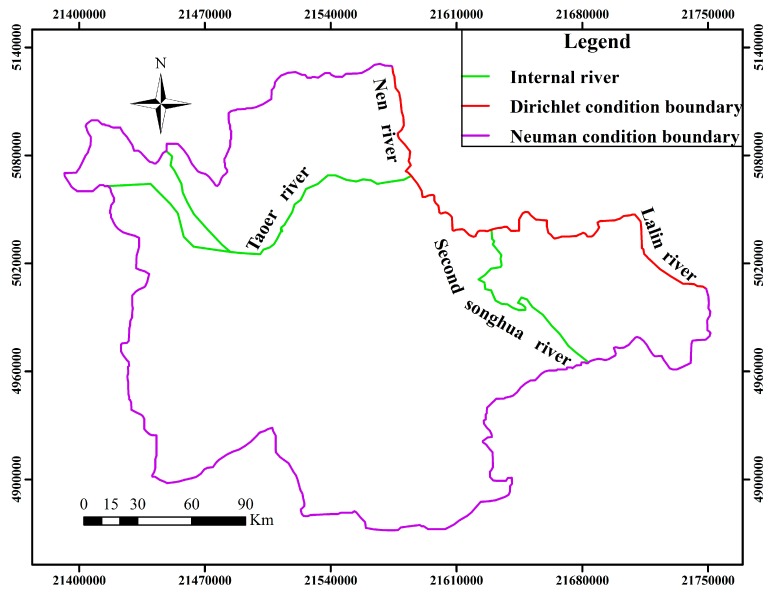
Types of lateral boundary in study area.

The top of the simulation area is the unconfined aquifer’s upper boundary where such actives pertaining to water exchange mainly occur as precipitation infiltration, irrigation leakage, evaporation, artificial exploitation, *etc*. The bottom boundary of the simulation area is the floor of the confined aquifer which is a clayey soil layer and almost has no water exchange. The lateral boundary is generalized based on the boundary of unconfined aquifer (Figure 1), because the unconfined aquifer is found relatively thicker.

The groundwater flow system of the simulation area can be generalized as non-homogeneous, isotropic, and two-dimensional unsteady flow system, which can be shown as follows [41,42]:

(16) $$\begin{array}{ll} \frac{\partial }{\partial x}k\left(H\left(x,\;y,\;t\right)-Z_{b}\right)\frac{\partial H\left(x,\;y,\;t\right)}{\partial x}+\frac{\partial }{\partial y}k\left(H\left(x,\;y,\;t\right)-Z_{b}\right)\frac{\partial H\left(x,\;y,\;t\right)}{\partial y}\\ \;+W=\mu \frac{\partial H\left(x,\;y,\;t\right)}{\partial t} & \cdots \cdots \left(x,\;y\right)\in D,\;t\geq 0\\ H\left(x,\;y,\;t\right){|}_{t=0}=H_{0}\left(x,\;y\right) & \cdots \cdots \left(x,\;y\right)\in D,\;t=0\\ H\left(x,\;y,\;t\right){|}_{{\text{Γ}}_{1}}=H_{1}\left(x,\;y,\;t\right) & \cdots \cdots \left(x,\;y\right)\in {\text{Γ}}_{1},\;t>0\\ k\left(H-Z_{b}\right)\frac{\partial H}{\partial \vec{n}}{|}_{{\text{Γ}}_{2}}=q\left(x,\;y,\;t\right) & \cdots \cdots \left(x,\text{​}\;y\right)\in {\text{Γ}}_{2},\;t>0 \end{array}$$

where *H*(*x*, *y*, *t*) is the groundwater table (m), *H_0_*(*x*, *y*) is the initial water table (m), Z*_b_* is the elevation of aimed for aquifer floor (m), *k* is the hydraulic conductivity (m·d^−1^), *μ* is the specific yield (dimensionless), *W* is the vertical recharge, discharge strength of unconfined aquifer (m·d^−1^), ${\text{Γ}}_{1}$ is the boundary of Dirichlet condition, ${\text{Γ}}_{2}$ is the boundary of Newman condition, *q* (*x*, *y*, *t*) is the recharge and discharge quantity of aquifer per unit width (m·d^−1^), $\vec{n}$ is the direction of outward normal on the boundary, *D* is the area for simulation computation.

**Figure 2:**
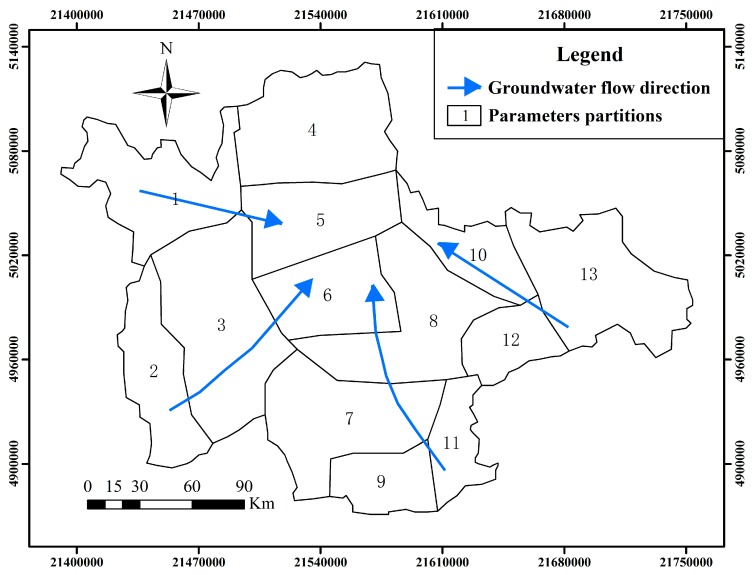
Groundwater flow direction and parameters partitions of study area.

The groundwater flow direction and parameters partitions of the study area are shown in Figure 2, in which the study area is divided into 13 subareas, and the parameters values of study subareas are in Table 1.

**Table 1:** Parameters values of study subareas.

Partitions Parameters	1	2	3	4	5	6	7	8	9	10	11	12	13
Hydraulic conductivity (m/d)	14	135	27	17	20	26	9	11	12	13	28	44	15
Specific yield	0.09	0.23	0.10	0.12	0.18	0.08	0.08	0.10	0.11	0.08	0.09	0.10	0.15
Specific storage (m^−1^)	0.008	0.008	0.009	0.008	0.008	0.008	0.009	0.008	0.008	0.008	0.007	0.008	0.008

The Groundwater Modeling System (GMS) is made of several modular (MODFLOW, FEMWATER, MT3DMS, RT3D and so on) designed by Environmental Model Laboratory of Brigham Yong University and Test Station of America Army Drainage Engineering. It was used to model groundwater flow and groundwater quality widely [43,44]. MODFLOW modular of GMS (version 9.2.2) software is used to solve the numerical simulation model of groundwater flow, and the algorithm of MODFLOW is a finite difference method [45,46,47].

#### 2.2.4. Genetic Algorithm

**Figure 3:**
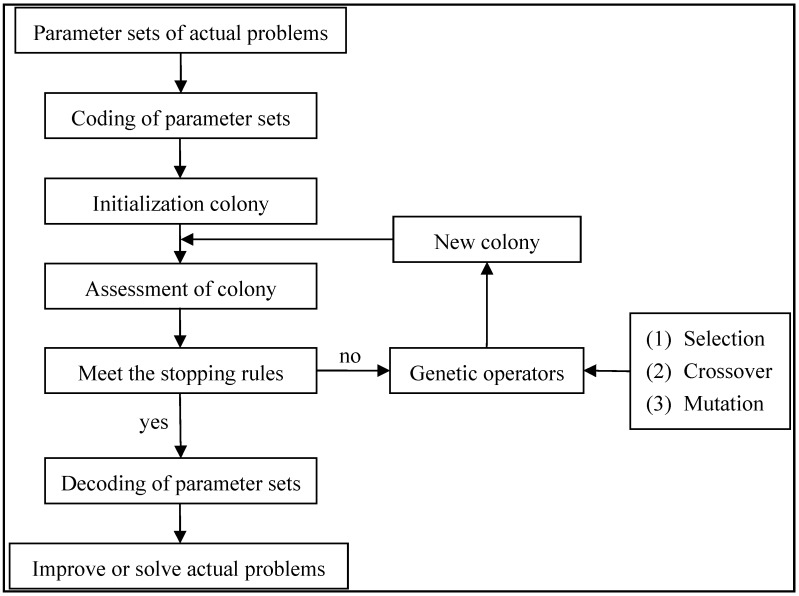
Process of the genetic algorithm.

The genetic algorithm (GA) is a computational model on the basis of Darwin's biological evolution theory genetic mechanism, used to search for the optimal solution by simulating natural evolution. It includes three genetic operators of selection, crossover and mutation [48,49,50]. A flowchart for solving a general problem through the genetic algorithm is shown in Figure 3.

## 3. Results and Discussions

### 3.1. Numerical Simulation of Groundwater Flow

**Figure 4:**
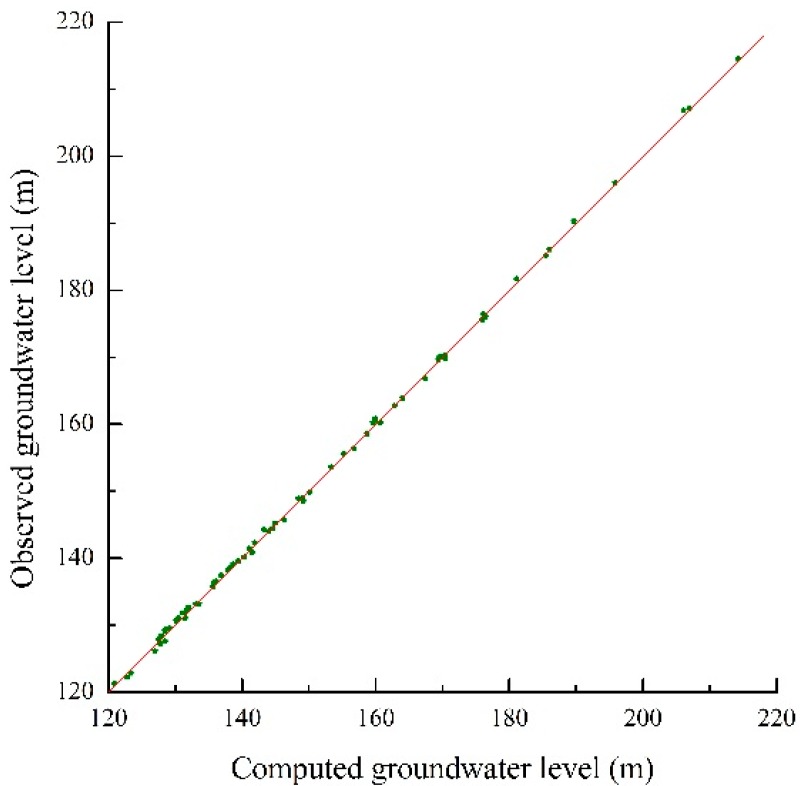
The fitting chart of the groundwater table between measured and computed of each observation well at the end of the model calibration stage.

**Figure 5:**
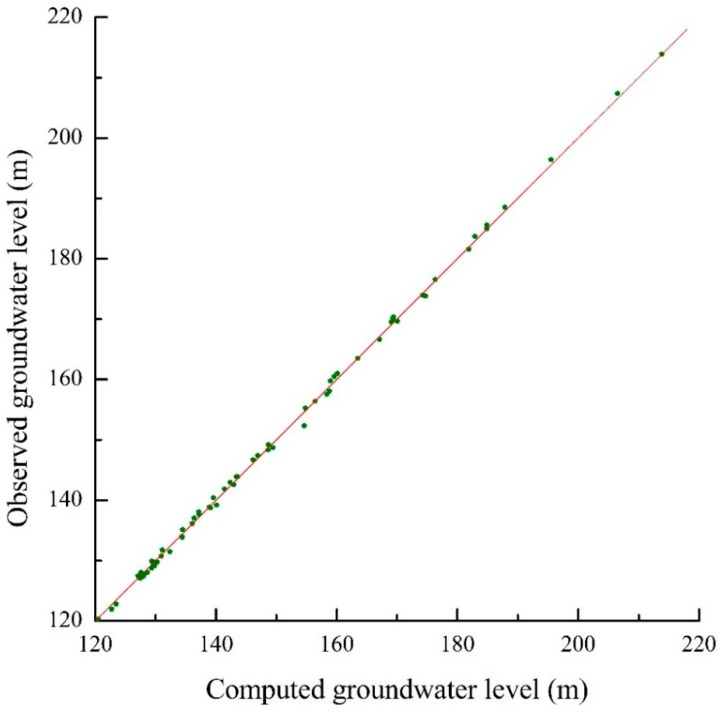
The fitting chart of the groundwater table between measured and computed of each observation well at the end of the model verification stage.

**Figure 6:**
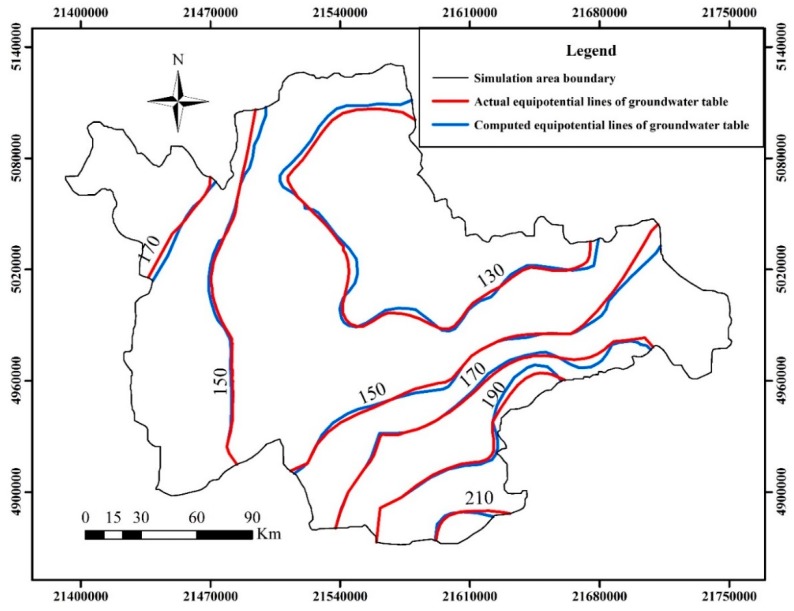
The actual and computed equipotential lines of groundwater table at the end of the model calibration stage.

**Figure 7:**
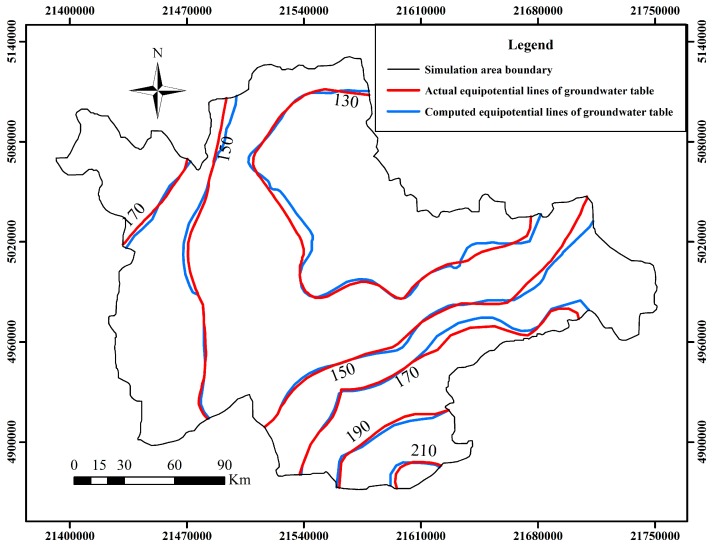
The actual and computed equipotential lines of groundwater table at the end of the model verification stage.

The calibration phase of simulation was selected in the dry season for 181 days from October 1, 2006 to March 31, 2007, taking into consideration that less source and sink are beneficial to identify hydrogeology parameters. The verification phase was selected in the wet season for 182 days from April 1, 2007 to September 30, 2007, on account that more source and sink are beneficial to verify the effectiveness of hydrogeology parameters. The fitting results of computed groundwater table and the actual measured groundwater table are shown in Figure 4 and Figure 5 at the end of the model calibration and verification stage respectively. The equipotential lines of the groundwater table are also shown in Figure 6 and Figure 7 at the end of the model calibration and verification stage respectively.

From Figure 4 and Figure 5 it can be seen that the slopes of the straight lines fitted by the actual measured groundwater table values and computed groundwater table values are all close to 1. From Figure 6 and Figure 7 it can be seen that the fitting results of equipotential lines between the actual groundwater table and the computed groundwater table are very good. The above description means that the actual measured groundwater table values are very close to the computed groundwater table values, the direction of computed groundwater flow field is in accordance with the actual groundwater flow field, the selected hydrogeological conceptual model generalization, partial differential equations and algorithm are reasonable and feasible, and the established numerical simulation model of groundwater flow can objectively and accurately describe the groundwater flow characteristics of the study area. The research results concluded above can give a good foundation for the establishment of a surrogate model.

**Figure 8:**
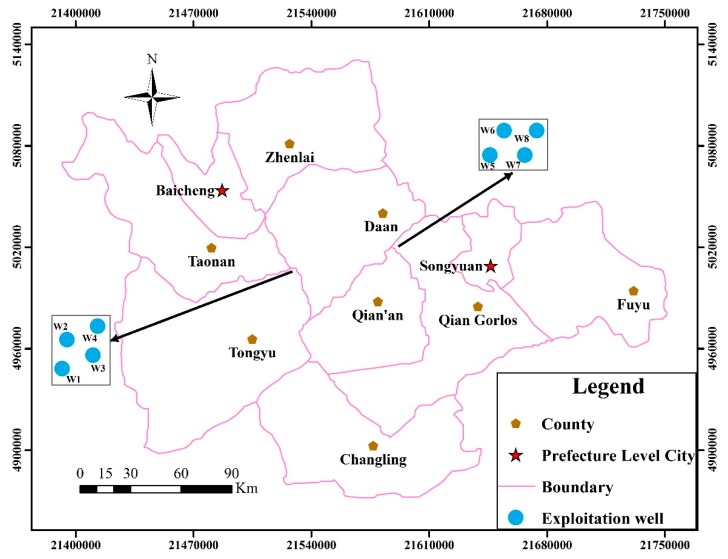
Exploitation wells distribution.

**Table 2:** Training and validation samples of surrogate model (*q*: m^3^/d, *s*: m).

Exploitation Scheme		$W_{1}$		$W_{2}$		$W_{3}$		$W_{4}$		$W_{5}$		$W_{6}$		$W_{7}$		$W_{8}$	
		q	s	q	s	q	s	q	s	q	s	q	s	q	s	q	s
Training samples	1	3240	0.808	7070	0.925	1551	0.609	2383	0.625	9949	1.848	8780	1.797	7840	2.395	1762	2.633
	2	5713	0.896	88	0.565	7889	0.967	1480	0.588	9378	1.814	6227	2.110	6331	2.451	6961	3.154
	3	1159	0.558	5852	0.799	1677	0.584	3829	0.743	9705	1.678	2826	1.380	6499	1.978	4905	1.969
	4	3786	0.844	3100	0.911	4463	1.016	6899	1.194	6296	1.324	2397	1.559	6756	1.963	7091	2.342
	5	9330	1.178	1742	0.823	367	0.732	4703	0.794	1990	0.956	5481	2.017	7740	2.162	7827	3.189
	6	3701	0.994	7724	1.151	3603	0.921	4171	0.958	1025	0.463	2156	0.830	4760	1.037	3034	1.304
	7	9759	1.732	7895	1.600	4834	1.413	6581	1.405	9128	1.722	9877	2.187	3480	2.242	4409	3.289
	8	204	0.395	838	0.535	6831	0.902	5248	0.960	1736	0.775	6610	1.449	7379	1.727	2606	2.280
	9	9174	1.262	2532	0.793	4706	0.854	393	0.451	4662	1.186	5797	2.007	4820	2.025	7566	3.110
	10	4479	0.933	3469	0.931	5121	1.035	5637	1.084	5798	1.213	9607	1.912	1892	1.755	3396	2.930
	11	6170	1.108	4151	0.851	7308	0.975	22	0.514	6172	1.354	9075	1.831	5945	2.073	2578	2.785
	12	2540	0.798	6649	1.051	1914	0.815	6084	1.080	3254	0.667	5078	0.852	2505	0.960	349	1.285
	13	7677	1.306	4740	1.200	3494	1.136	6789	1.250	3793	0.893	7353	1.603	1648	1.427	3701	2.483
	14	4990	0.896	4252	0.836	2507	0.731	3374	0.726	2265	0.548	703	1.059	606	0.890	6730	1.647
	15	4106	0.919	7451	1.081	71	0.680	4881	0.903	2559	0.701	4057	1.474	1147	1.236	6304	2.307
	16	477	0.497	5428	0.664	4359	0.597	964	0.469	7196	1.354	7816	1.443	5053	1.776	981	2.135
	17	1275	0.404	1098	0.399	5253	0.630	1961	0.508	8612	1.219	162	0.777	253	0.963	4337	1.032
	18	1887	0.562	2336	0.583	5738	0.773	2653	0.661	5325	1.135	9330	1.704	2898	1.679	2037	2.607
	19	7164	1.123	3718	1.053	710	0.907	7674	1.209	8122	1.711	8556	2.165	7467	2.529	5020	3.273
	20	6440	1.314	7240	1.238	6091	1.143	2830	0.916	7733	1.605	6778	1.956	7181	2.342	5364	2.946
	21	2993	0.683	2633	0.643	5531	0.796	2437	0.652	5137	0.935	345	0.861	4008	1.186	4615	1.250
	22	4699	0.701	1290	0.568	2016	0.610	3710	0.653	2144	0.621	3799	0.787	6022	1.171	696	1.205
	23	1570	0.510	2824	0.632	3314	0.682	4471	0.802	4863	1.104	4859	1.607	4583	1.738	5564	2.459
	24	8262	1.050	277	0.794	518	0.826	7581	1.100	561	0.398	3118	1.033	2647	0.970	4012	1.646
	25	6686	1.173	3845	1.103	3868	1.118	7098	1.261	7271	1.449	3308	1.575	6944	2.037	6132	2.344
	26	2492	0.802	5630	0.867	6446	0.896	1652	0.675	6907	1.316	5608	1.403	5268	1.753	2898	2.077
	27	2240	0.556	3392	0.684	1352	0.596	5016	0.819	7944	1.104	1088	0.486	1516	0.841	1122	0.578
	28	8070	1.443	6986	1.430	2211	1.175	7823	1.403	196	0.537	4293	1.689	3336	1.465	7617	2.712
	29	8956	1.439	4502	1.037	7600	1.144	401	0.625	2839	0.786	8151	1.703	986	1.393	3895	2.671
	30	8525	1.266	2056	0.832	7166	1.065	1217	0.629	3511	0.862	7065	1.152	5628	1.452	7	1.759
	31	7758	1.252	760	0.997	7616	1.387	7268	1.355	348	0.447	2552	1.385	2193	1.163	7287	2.218
	32	5984	1.025	5310	0.979	984	0.734	4325	0.832	1283	0.548	8428	1.450	1298	1.173	1916	2.301
	33	979	0.588	6563	0.915	1058	0.641	5425	0.937	8359	1.330	7649	1.371	18	1.340	1324	1.995
	34	532	0.537	4979	0.850	2987	0.780	6210	1.058	3206	0.929	4557	1.581	5536	1.725	5745	2.458
	35	5259	0.848	1951	0.674	4033	0.778	3028	0.678	4433	0.988	6495	1.512	3125	1.524	3543	2.319
	36	6849	1.162	6251	0.994	2756	0.758	1174	0.550	4163	0.888	884	1.174	3674	1.327	6595	1.780
	37	5164	0.838	554	0.627	5950	0.919	3516	0.772	5687	0.916	1485	0.479	4378	0.981	501	0.621
	38	7293	1.311	6169	1.084	6217	1.034	755	0.641	6593	1.150	3712	1.427	561	1.343	5830	2.129
	39	3372	0.700	1530	0.582	6783	0.861	2087	0.630	851	0.294	1719	0.513	2310	0.596	1572	0.801
	40	9738	1.513	5192	1.275	3026	1.139	5848	1.157	8827	1.363	1888	0.820	3998	1.331	2248	1.091
Validation samples	1	2032	0.512	952	0.573	4167	0.783	5642	0.932	2861	0.626	1031	0.842	2757	0.956	4332	1.279
	2	5050	1.079	6829	1.137	3100	0.926	4497	0.971	1064	0.647	2940	1.280	7119	1.556	5172	2.026
	3	553	0.346	4431	0.487	1193	0.304	866	0.300	6786	1.623	9843	2.472	7295	2.662	6212	3.804
	4	6490	1.368	7557	1.311	6734	1.237	3334	1.015	9399	1.520	5333	1.162	4142	1.606	1232	1.628
	5	4363	1.079	6000	1.156	6113	1.167	5259	1.153	8178	1.242	4296	0.886	1879	1.160	791	1.217
	6	1630	0.475	3426	0.561	2283	0.503	2514	0.535	5859	0.997	3302	1.054	1484	1.132	3431	1.547
	7	9206	1.509	5357	1.134	7365	1.181	759	0.690	738	0.766	7649	2.126	5331	1.966	7055	3.400
	8	3615	0.786	2909	0.869	3417	0.932	7145	1.175	3935	0.823	6468	1.390	448	1.182	3200	2.137
	9	8124	1.097	1635	0.904	100	0.839	7783	1.148	7514	1.203	134	0.591	5865	1.275	2166	0.753
	10	7193	0.970	118	0.571	5252	0.822	1685	0.531	4985	1.237	8026	2.241	3244	2.062	7281	3.481

### 3.2. Surrogate Model of Numerical Simulation Model of Groundwater Flow

The four exploitation wells were set in the Tongyu county and Qian Gorlos county respectively so as to supply water to Daan county shown in Figure 8. The Latin hypercube sampling method was used to obtain 40 and 10 groups of exploitation schemes which were introduced into the numerical simulation model of the groundwater flow to obtain groundwater table drawdown datasets respectively (Table 2).

The *q* is the groundwater exploitation quantity and *s* is the groundwater table drawdown under the exploitation schemes in Table 2.

MATLAB (2013a) procedure was compiled according to the principle of the regression kriging method. Training samples were used to establish the surrogate model (regression kriging model) and validation samples were used to verify the computational accuracy of the surrogate model. The θ are a series of coefficients of gauss functions which determine the precision of the surrogate model. The θ are calculated by a genetic algorithm through Equation (16) in Table 3.

**Table 3:** Parameters of the surrogate model.

Parameter	$\theta_{1}$	$\theta_{2}$	$\theta_{3}$	$\theta_{4}$	$\theta_{5}$	$\theta_{6}$	$\theta_{7}$	$\theta_{8}$
Value	0.7922	0.9961	0.5000	1.6490	0.6476	0.4952	0.5737	1.0513

**Figure 9:**
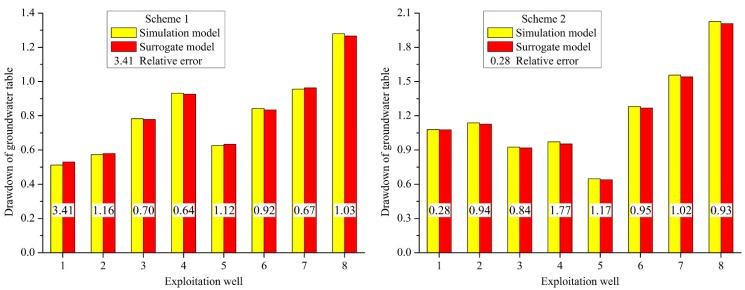

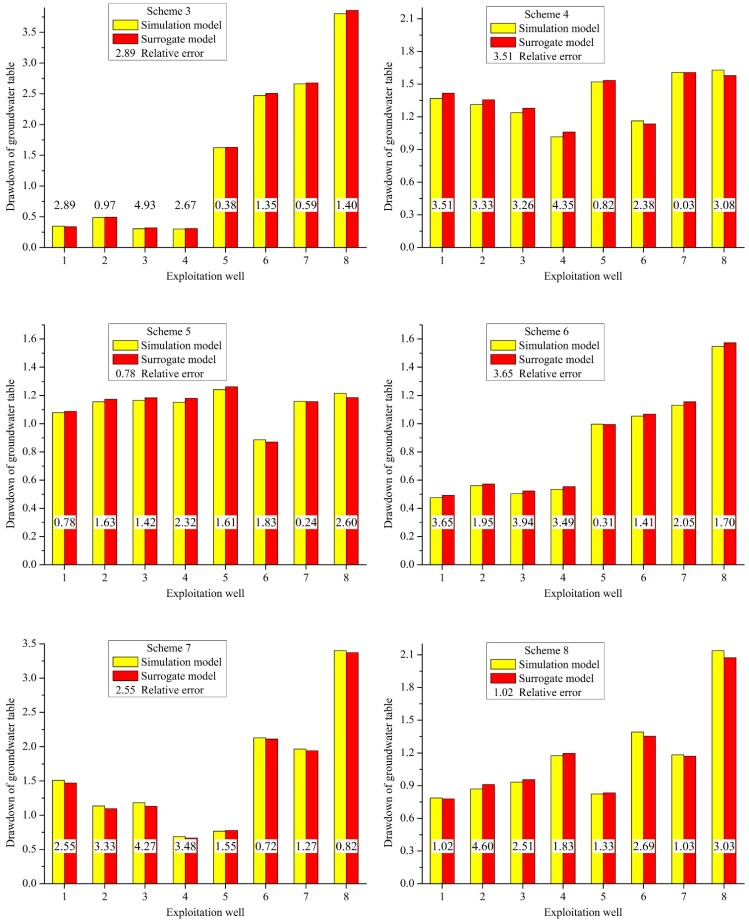

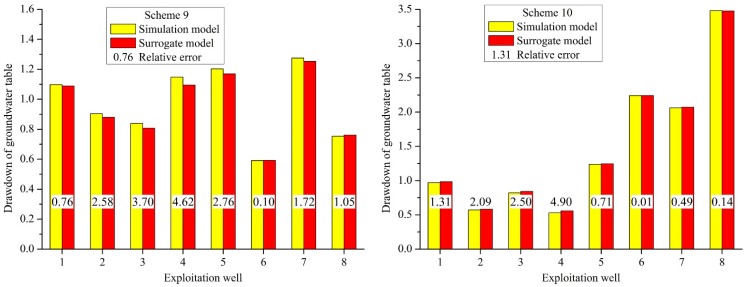
Value and relative error of groundwater table drawdown of the simulation model and surrogate model.

To investigate the validity of the surrogate model, the validation samples were introduced into the groundwater flow numerical simulation model and the surrogate model respectively. Then, the results of the surrogate model and the numerical simulation model of the groundwater flow were compared with the evaluation indexes including relative error and root mean square error. The value and relative error of the groundwater table drawdown of the simulation model and surrogate model are shown in Figure 9, the mean relative error and root mean square error between the simulation model and the surrogate model are shown in Table 4.

From Figure 9, the relative error of each well of each scheme is between 0.01% and 4.93%, less than 5%, and the mean relative error of each scheme is between 0.99% and 2.60%. The mean relative error of the 10 validation schemes is 1.87%, which shows that the computed groundwater table drawdown of each well of each scheme by the kriging model is very close to the simulation model. The root mean square error of each scheme is between 1.06% and 2.93%, and the root mean square error of the 10 validation schemes is 2.27%. The results show that the computed groundwater table drawdown of each well of each scheme by the kriging model is not significantly different close to the simulation model, and each scheme also has no significant difference. The above description demonstrates that the surrogate model could substitute the groundwater flow numerical simulation model effectively.

**Table 4:** The mean relative error and root mean square error between the simulation model and surrogate model.

Scheme	Mean Relative Error		Root Mean Square Error	
1	1.21	1.87	1.48	2.27
2	0.99		1.06	
3	1.90		2.37	
4	2.60		2.93	
5	1.55		1.71	
6	2.31		2.60	
7	2.25		2.58	
8	2.26		2.53	
9	2.16		2.60	
10	1.52		2.15	

### 3.3. Optimization Model

Considering that the eight pumping wells were used to supply water for Daan county water simultaneously, the exploitation quantity of each well needed to be distributed reasonably according to the minimum average groundwater table drawdown of the eight exploitation wells. However, the cost of water supply of each exploitation well is different. We selected an optimal exploitation scheme which could make the average drawdown of the groundwater table and the cost of the groundwater exploitation less.

To optimize the conditions of water supply for the scheme of groundwater exploitation, a nonlinear multi-objective optimizations model was developed using the minimum average drawdown of the groundwater table and the minimum cost of groundwater exploitation as multi-objective functions, with the exploitation rates as decision-making variables. The optmization model was constructed as follows:

(17) $$\left\{ \begin{array}{l} S=\frac{1}{n}\sum_{i=1}^{n}s_{i}\\ M=\sum_{i=1}^{n}x_{i}\cdot q_{i} \end{array}\right.$$

(18) $$\text{Subject to: }\left\{ \begin{array}{l} s_{i}=f(q_{i})\\ 0\leq q_{i}\leq 8000,\;\left(i=2,\;3,\;4,\;7,\;8\right)\\ 0\leq q_{i}\leq 10000,\;\left(i=1,\;5,\;6\right)\\ \sum_{i=1}^{8}q_{i}\geq 60000 \end{array}\right.$$

where *S* is the average groundwater table drawdown (m), *s_i_* is the groundwater table drawdown of the *i^th^* well (m), n is the numbers of exploitation wells, *M* is the water cost ($), *x_i_* are cost coefficients in the Table 5 ($·d·m^-3^), *q_i_* are the exploitation rates of the $i^{th}$ well (m^3^ d^−1^).

**Table 5:** Water cost coefficients ($·d·m^-3^).

Well	$x_{1}$	$x_{2}$	$x_{3}$	$x_{4}$	$x_{5}$	$x_{6}$	$x_{7}$	$x_{8}$
Cost coefficient	2	2	2	2	3	3	3	3

In order to solve the nonlinear multi-objective optimization model, the surrogate model (regression kriging program) was loaded into the genetic algorithm and linked with the exploitation rates. The optimal groundwater exploitation strategy through invoking the surrogate model is in the Table 6.

**Table 6:** The optimal exploitation scheme.

Exploitation Well	$W_{1}$	$W_{2}$	$W_{3}$	$W_{4}$	$W_{5}$	$W_{6}$	$W_{7}$	$W_{8}$
Exploitation quantity (${\text{10}}^{3}\cdot {\text{m}}^{3}\cdot {\text{d}}^{-1}$)	7.597	7.585	7.737	7.592	7.593	7.724	7.585	7.596
Groundwater table drawdown (m)	0.400	0.411	0.422	0.426	0.427	0.607	0.675	0.929
Water cost ($10^{3}\cdot \$\cdot d\cdot {\text{m}}^{-3}$)	15.194	15.170	15.474	15.184	22.779	23.172	22.755	22.788

The main computational burden of the simulation optimization process is the repeated running of the numerical simulation model. The optimization for the study area requires 55 s of CPU time to run every simulation model on a 2.93 GHz Inter CPU and 2 GB RAM PC platform. A conventional simulation optimization model requires 40000 runs of the simulation model. Thus, it would require 2200000 s (25 days) of CPU time to process the overall simulation model run. However, in this study replacing the simulation model with the surrogate model in the optimization process could reduce the process to only 50 simulation model runs during the training and validation of the surrogate model. Thus, it only required 19800 s (5.5 h) of CPU time to complete the simulation model and optimization model run.

## 4. Conclusions

(1) The groundwater table values calculated by the numerical simulation model of groundwater flow are very close to the actual measured values both at the stage of model calibration and model verification, which demonstrates that the selected hydrogeological conceptual model generalization, partial differential equations and algorithm are reasonable and feasible in the study area, and the established numerical simulation model of groundwater flow can objectively and accurately describe the groundwater flow characteristics of the study area. These research results can provide a good foundation for establishing a surrogate model.
(2) Due to the regression kriging method with accurate approximation ability, the surrogate model results are much closer to that of the numerical simulation model of groundwater flow, and could effectively substitute the numerical simulation model of groundwater flow.
(3) The huge computational burden of coupled operations during simulation and optimization hinders the success of the simulation optimization model in groundwater exploitation. According to this study, replacing the simulation models with surrogate models could reduce the huge computational burden effectively and maintain considerably high accuracy so as to obtain an optimal exploitation scheme.
